# Elder abuse in Europe’s “most elderly” city: an update of the phenomenon based on the cases reported to the Penal Court of Genoa from 2020 to 2023 and literature review

**DOI:** 10.1007/s40520-025-03153-x

**Published:** 2025-08-08

**Authors:** Martina Drommi, Rosario Barranco, Francesco Ventura, Andrea Molinelli

**Affiliations:** 1 Department of Legal and Forensic Medicine, Health Science Department (DISSAL), University of Genoa, via De Toni 12, Genoa, 16132 Italy; 2 IRCCS-Ospedale Policlinico San Martino Teaching Hospital, Largo Rosanna Benzi 10, Genoa, 16132 Italy

**Keywords:** Elder abuse, Domestic violence, Personal injury, Prosecutor’s office, Sentence

## Abstract

**Background:**

Elder abuse is a global problem, with literature indicating that one in six elderly individuals is a potential victim.

**Aims:**

In this study, we delve into reported cases of elder abuse brought to the attention of the Judicial Authority in the Genoa area between 2020 and 2023. Our objective is twofold: first, to conduct a detailed analysis of epidemiological data to quantify the incidence and characteristics of this phenomenon; second, to critically examine the medico-legal and clinical aspects emerging from these cases, aiming to contribute to a deeper understanding and improved management of this social and health issue.

**Methods:**

We analysed data on reports of abuse processed by the Court of Genoa from 2020 to 2023. These findings were then compared with data from the previous 10-year period and with existing literature.

**Results:**

A total of 1011 reports related to offenses concerning violations of family assistance obligations, abuse of means of correction or discipline, maltreatment of family members and cohabitants, personal injury, abandonment of minors or incapacitated persons, and circumvention of incapacitated persons were identified. Among 24,144 judgments, 200 relevant to potential elder abuse situations were examined.

**Discussion:**

The findings indicate that abuse was predominantly perpetrated within the domestic setting and primarily by the victims’ relatives. Key risk factors identified included the victim’s female gender, their dependency on others, and the perpetrator’s concurrent mental illness or substance abuse.

**Conclusion:**

Elder abuse, therefore, constitutes an extremely complex and heterogeneous problem, presenting an infinite number of manifestations and facets, making it difficult to perceive and identify.

## Introduction

Elder abuse is an extremely current and significant phenomenon within our society. It is estimated, in fact, that globally 1 in 6 elderly individuals is a victim of some type of abuse, a numerical datum corresponding to approximately 141 million victims per year among those over 60 [1].

According to the World Health Organization, Elder Abuse is defined as “A single or repeated act, or lack of appropriate action, occurring within any relationship where there is an expectation of trust, which causes harm or distress to an older person (conventionally aged 60 years or older). The definition includes physical, sexual, psychological and emotional abuse; financial and material abuse; abandonment; neglect; and serious loss of dignity and respect” [2].

The definition provided by the National Center on Elder Abuse (NCEA) frames mistreatment as “an intentional act, or failure to act, by a caregiver or another person, in a relationship involving an expectation of trust” [3].

Physical or sexual assaults, financial exploitation, and verbal/psychological abuse are considered commission behaviors, while neglect and disregard are omission behaviors. Generally, the phenomenon is closely associated with situations of psychological and social distress, as well as an increase in morbidity and mortality of the elderly, with a consequent increase in the number of emergency room visits and hospitalization rates [4].

According to the World Health Organization (WHO), we can distinguish two broad macro-categories of abuse: abuse in the domestic (community) setting [1] and abuse in institutional care settings (nursing homes, hospitals) [5].

Regarding abuse in the domestic setting, the phenomenon of mistreatment seems to affect 15.7% of subjects over 65; concerning the various types of abuse, the following percentages are reported: 11.6% for psychological abuse; 6.8% for financial abuse; 4.2% for neglect (abandonment and disregard); 2.6% for physical abuse; 0.9% for sexual abuse. Regarding abuse in institutional care settings, they show a higher percentage of events (approximately 2 out of 3 staff members report having committed some type of mistreatment) [6].

The frail elderly can also be subject to an extremely insidious form of abuse, namely verbal or psychological abuse which, despite leaving no macroscopic clues, can generate potentially devastating consequences for the victim. This type of mistreatment consists of causing anguish, pain, or psychological discomfort through verbal language or non-verbal acts. It therefore includes any type of verbal aggression, threat, intimidation, or harassment, as well as the forced isolation of the victim from loved ones and their removal from regular activities. The prolonged psychological distress to which this type of violence leads can result in mood changes, up to frank depression, with a significant decrease in quality of life [7]. The concept of elder abuse also includes omission behaviors (i.e., refusal or negligence on the part of the caregiver in the duty of assistance and care).

As for the perpetrators of mistreatment, they are, in fact, most frequently family members, forced to assume the role of caregivers; this occurs predominantly in the community setting, while in institutional care contexts (nursing homes, care homes, etc.), healthcare professionals themselves assume the role of caregivers and, therefore, of potential perpetrators of mistreatment [6].

In the literature, numerous victim-specific factors have been identified that can facilitate the onset of abuse situations, such as the elder’s functional and physical dependence on the caregiver. The presence of physical limitations, disabilities, dementia, or poor physical or mental health conditions, places the individual in a condition of greater vulnerability and makes them predisposed to becoming a victim of abuse [8].

More controversial is the influence of demographic factors such as gender, age, and ethnicity, which are more affected by the variability linked to the sociocultural and geographical context in which they are analyzed [9].

In relation to age, some studies report that abuse is more often perpetrated against younger elderly individuals; the “young old,” in fact, often live in the same household with their spouse or children, who are the main perpetrators of mistreatment [10].

Finally, among the predisposing causes of abuse related to the victim’s profile are a history of past suffered violence, aggressive behavior on the part of the elderly person, a positive history for psychiatric problems or substance abuse, and social isolation [6].

The risk factors related to the caregiver as a perpetrator of violence against the elderly are also numerous and heterogeneous. The main ones include a past or present history of drug abuse, a positive history for psychiatric disorders or physical problems, social isolation, the existence of financial problems (or unemployment) that can make the individual dependent on the victim, and finally the perception of an excessive burden of responsibility [10].

In Italy, there is still no specific law to protect the elderly. Consequently, violence against them is managed through articles already present in the Penal Code. The most pertinent are Article 570 (Violation of family assistance obligations), Article 571 (Abuse of means of correction or discipline), Article 572 (maltreatment of family members and cohabitants), Article 582 (Personal injury), Article 591 (Abandonment of minors or incapacitated persons), and Article 643 (Circumvention of incapacitated persons).

Due to their inherent fragility, the elderly are particularly exposed to these crimes. Their consequences, in terms of prognosis, represent a serious social and economic problem for the entire society.

In this study, we delve into reported cases of elder abuse that have been brought to the attention of the Judicial Authority in the Genoa area between 2020 and 2023. Our objective is twofold: on the one hand, to conduct a detailed analysis of epidemiological data to quantify the incidence and characteristics of the phenomenon; on the other hand, to critically examine the medico-legal and clinical aspects emerging from these cases, in order to contribute to a deeper understanding and better management of this social and health issue.

## Materials and methods

Our study examined cases of elder abuse (individuals aged over 65 years, both male and female) brought to the attention of the Judicial Authority in the metropolitan area of Genoa between 2020 and 2023. To conduct this analysis, we obtained data for the 2020–2023 period directly from the Public Prosecutor’s Office. Reports and judgments from the analysed period were examined.

The inclusion criteria were characterized by the reporting of abuse offenses (violation of family assistance obligations, abuse of means of correction or discipline, maltreatment of family members and cohabitants, personal injury, abandonment of minors or incapacitated persons, and circumvention of incapacitated persons) and the victim’s age exceeding 65 years.

We must clarify that the number of reports does not correspond to the number of judgments. Reports brought to the attention of the Judicial Authority in 2023 may not yet have gone to trial; similarly, not all reports are deemed worthy of going to trial, and their judicial process concludes earlier (dismissal, withdrawal of complaint, etc.). Furthermore, judgments issued during the four-year period under examination may have concerned reports from previous years.

The numbers of reports, therefore, were acquired by us for completeness in the analysis of the phenomenon, while the conclusions we reached were derived solely from the reading of the merits judgments.

These data were then compared with the results of previous research conducted by the Institute of Legal Medicine at the University of Genoa, which had already examined case studies relating to the two preceding five-year periods (2010–2015 and 2015–2019) [11, 12]. This approach allowed us to outline a current picture of the phenomenon and trace its evolution over the last 14 years, providing an essential historical perspective. Through careful reading and analysis of the content of judgments issued by the Ordinary Court of Genoa, crucial information regarding the different types of elder abuse was extrapolated. Specifically, we identified: the sex of the injured party (the victim of the crime); the relationship between the perpetrator and the victim; the locations where the mistreatment occurred, providing indications of higher-risk contexts; and the outcomes of the pronounced judgments (conviction or acquittal), which offer an overview of the judicial response to these crimes.

This in-depth examination allowed us to delineate not only the characteristics of the abuse, but also the profiles of both abusers and victims, including details on sex, age, psychological profile, and any pathologies of both.

## Results

A total of 1011 reports were identified, concerning offenses related to: violation of family assistance obligations, abuse of means of correction or discipline, maltreatment of family members and cohabitants, personal injury, abandonment of minors or incapacitated persons, and circumvention of incapacitated persons (Table 1). In total, 24,144 judgments were examined, and among these, 200 were found to be related to potential elder abuse situations (0.82%). Overall, out of a total of 182 abuse victims (in some instances, the same elderly individual was subjected to multiple types of abuse), 80 were male and 102 were female. A significant disparity between the two genders was observed for the offenses of " maltreatment against family members " and “personal injury.” In the former, women were confirmed as predominantly at risk of abuse (51 women vs. 23 men), while in the latter, the male gender more frequently became the victim of the crime (37 men vs. 23 women). Regarding the perpetrators of abuse: out of a total of 201 defendants (as with victims, some judgments involved multiple active subjects of the crime), 121 were male and 80 were female. Considering the individual types of offenses, clear differences between the two genders were found in relation to the crimes of “ill-treatment against family members and cohabitants” and “personal injury” compared to the crime of “circumvention of incapacitated persons.” While in the first two categories, the male gender of the perpetrator of abuse was clearly prevalent, in the third category, out of a total of 57 defendants, 23 were men and 34 were women, consequently confirming that elder abuse perpetrated by females more frequently takes on the characteristics of deception, with the aim of obtaining financial benefit. The elderly person’s home emerged as the location where the highest number of abuses occurred (78% of cases). Only 6 cases of suspected abuse took place in elderly care homes, representing “only” 3% of the locations of mistreatment; the remaining 19% of abuse situations occurred in public places (condominiums, on the street, at a bar, on public transport such as trains or buses, etc.). It must be highlighted that regarding the cases that occurred in elderly care homes, 4 ended with the acquittal of the defendants, while only 2 processes concluded with a conviction.

**Table 1:** Analysis of the reports collected in Genoa during the period 2020–2023

	2020	2021	2022	2023	TOTAL
**Art 570 - violation of family care obligations**	9	6	1	3	19
**Art 571 - abuse of the means of correction and discipline**	0	0	1	1	2
**Art 572 - maltreatment of family members**	70	57	53	49	229
**Art 582 - personal injury**	147	147	123	136	553
**Art. 591 - abandonment of minors or incapacitated persons**	11	7	8	8	34
**Art. 643 - circumvention of incapable persons**	35	69	25	35	164
**Art. 609 bis - sexual violence**	2	5	0	3	10
**TOTAL**	274	291	211	235	1011

More specifically, during the four-year period under examination, a total of 19 crime reports related to “violation of family assistance obligations in the elderly” were received. In the same timeframe, only 2 judgments were issued concerning legal disputes over the non-payment of alimony between ex-spouses over 65 years of age. In no case was an abuse situation detected.

Regarding the offense of “abuse of means of correction and discipline,” 2 crime reports were found, one in 2022 and one in 2023. This is an offense whose physical signs, related to beatings, personal injuries, or evidence of forced restraint, are visible on the victim’s body for a certain period of time and, if promptly reported, lend themselves to a rather easy search for the necessary evidence to confirm the criminal conduct. The judgments related to this type of offense in the analysis period were 3, and 2 cases concerned nursing homes for the elderly. All cases concluded with the acquittal of the defendants.

Concerning the offense of “maltreatment against family members and cohabitants,” the total number of crime reports was 229 involving elderly individuals. In parallel, the judgments issued by the Court in the same timeframe constituted a significantly lower number, totalling 65. All analysed cases occurred within the domestic environment. In 12 out of 65 cases, the financial component of the mistreatment was found (pecuniary demands made against the victim). Finally, in 6 cases, among the harassing behaviours adopted by the defendant, the forced isolation of the victim from their loved ones was reported. In 74% of the examined cases, the perpetrator of the mistreatment was identified as the victim’s child. A smaller proportion (20%) was represented by the spouse, while an even smaller percentage (6%) was by caregivers. Out of a total of 65 analysed cases, 43 defendants were convicted and 22 acquitted. In 10 of the 22 cases where the defendant was acquitted, the abuse and mistreatment had indeed occurred, but the perpetrator was nevertheless acquitted due to “total mental incapacity.”

Regarding the crime reports of “personal injury” against an individual over sixty-five years of age, the total amounted to 553. The number of judgments for the period under review was 85. 48 cases related to physical mistreatment of the elderly occurring in a domestic setting (in 20 cases, the crime of personal injury was associated with the crime of family mistreatment); only one case occurred in elderly care home while the remaining 36 judgments identified conflict situations outside the family context. Analysis of the judgments showed that in 48 cases, a criminal conviction was reached.

34 reports concerned “abandonment of minors or incapacitated persons.” These cases originated from intra-family conflict dynamics, where the presence of a non-self-sufficient elderly person generated problems among children who did not guarantee adequate care. In the period under review, 8 judgments were examined concerning six cases at home (with the investigated party being the children and, in one single case, the caregiver), one case occurred within an elderly care facility, and a final case occurred on a means of transport. With the exception of the latter case, in all analysed judgments, the investigated party faced criminal conviction.

164 crime reports concerned “circumvention of incapacitated persons.” These cases involved financial mistreatment of the elderly subject. In these instances, a genuine frailty syndrome correlated with significant depressive symptoms emerged, making the individual more susceptible to manipulation. A total of 36 judgments were found, and out of a total of 37 victims (in one case, the injured party of the crime consisted of two elderly spouses), 26 (70%) were over 85 years old, 7 (19%) were between 75 and 84 years old, and 4 (11%) were between 65 and 74 years old. In none of the examined cases was the perpetrator identified as the victim’s child. In only one case (3%), the circumvention occurred in a bar frequented by the victim, and in 5 cases (14%) in an elderly care home, with the remaining cases occurring at the elderly person’s home. Analysis of the judgments revealed that in 18 cases (half), the investigated parties were convicted.

In the period under review, 10 reports concerned “sexual violence” against an elderly person, and only one judgment was found. The case concluded with an acquittal due to insufficient evidence: it was not possible to precisely identify the perpetrator of the crime.

In conclusion, out of a total of 170 judgments examined (in some cases, multiple charges were ascribed to the same defendant), 100 resulted in the conviction of the perpetrator, while in 70 cases, the perpetrator was acquitted. However, acquittal was not always “full”: in 2 cases, the perpetrator was acquitted due to death, in 14 cases, they were acquitted due to “total mental incapacity,” in 10 cases, they were acquitted due to withdrawal of complaint, and in 6 cases, they were acquitted because the crime had expired due to statute of limitations. Full acquittal of the defendant after due process was obtained in only 38 cases.

## Discussion

Despite the problem of elder abuse having gained extreme relevance over the years, and its dimensions being inevitably destined to increase, elder abuse remains an underestimated phenomenon; it is believed, in fact, that it is reported to the competent authorities in only 1 out of 24 cases [13].

The reasons why the phenomenon largely remains submerged are to be found primarily in the relational dynamics that are established between the caregiver and the assisted person. A first factor to consider is the fear of possible retaliation and further abuse by the aggressor, as well as possible abandonment or institutionalization [14].

A certain difficulty on the part of the elderly person in reporting what has happened exists. This reticence is mainly attributable to psychological factors: the lack of awareness of being a victim of abuse, a perverse sense of gratitude towards the caregiver, self-blame for what has happened, or even a profound sense of shame in admitting to not having been able to protect their own safety. Many hospitals, moreover, suffer from the lack of protocols for the identification and subsequent management of the problem, and staff often lack adequate training in this area [15].

According to our study, a consistent prevalence of conviction of the defendant is found only for the offenses of " maltreatment against family members and cohabitants” and “personal injury.”

Considering, instead, the offense of abuse of means of correction or discipline, acquittals of the defendant on all charges prevail, albeit with a minimal difference (2 acquittals and 1 conviction), while for the offense of abandonment of minors or incapacitated persons, convictions prevail, albeit in small numbers (5 convictions and 3 acquittals).

Finally, for the crime of Circumvention of incapacitated persons, the situation appears absolutely balanced, with 18 acquittals (for various reasons) and 18 convictions.

We must emphasize that the penalties faced by the defendants in question are often minimal, consisting of one or at most two years of imprisonment, along with the payment of legal costs.

Regarding acquittals, however, it is often found how difficult it is, during the investigation, to find evidence that implicates the culprit with that degree of probability “close to certainty” characteristic of the criminal process.

Often, in fact, in crimes so difficult to prove, such as abuse of means of correction or discipline and circumvention of incapacitated persons, one finds oneself in extremely ambiguous situations.

The present study has shown that the most frequent place where elder abuse occurs is their own home, and only rarely does it happen in nursing homes. The reasons for this aspect are to be sought primarily in the socio-cultural changes concerning the elderly subject: with progressive aging and the consequent physical and/or cognitive decline, the elderly person ends up spending most of their time inside their own home.

Under such conditions, moreover, the presence of third parties (family members, care staff, etc.) often becomes necessary to cover the elderly person’s care needs, and on whom the latter becomes completely dependent. Similarly, very often, a state of forced cohabitation of the elderly with their children becomes indispensable. This situation, in the long term, can become a source of mutual stress and discomfort, a condition that increases the probability of violent behavior or actual mistreatment against the elderly victim.

Elderly care homes, contrary to what is often reported in the media, are configured instead as the “safest” place for the assistance of the elderly subject and represented 3% of the places of mistreatment.

Comparing the data of the present study with previous data [11, 12], in the five-year period 2020–2023, a total of 200 judgments meeting the inclusion criteria for the investigation came to our attention, a significantly higher number compared to the 63 cases found in the five-year period 2010–2014 and, also, to the 156 cases found in the five-year period 2015–2019.

The number of reported complaints also confirms this trend, with 1011 crime reports related to the four-year period 2020–2023 examined, compared to 979 crime reports related to the five-year period 2015–2019 and, especially, to 323 crime reports received by the Public Prosecutor’s Office at the Court of Genoa in the five-year period 2010–2014.

This is a particularly interesting finding: the basis for such an increase could, in fact, be an expression of greater public awareness of the problem. Regarding the enormous growth in the number of crime reports found in the 2020–2023 period, it is necessary to point out that this period was characterized by the COVID-19 pandemic.

As for the crime of " maltreatment against family members and cohabitants,” the total number of crime reports where the injured party was over sixty-five years old was 180 in the COVID three-year period, an increase of 38% compared to the previous three-year period.

It can therefore be hypothesized that the period of “forced” cohabitation among family members during the pandemic may have unmasked latent situations of abuse and repeated mistreatment over time, particularly threats and psychological abuse, often escalating into episodes of overt violence, with access to the Emergency Room and reporting of the event by healthcare professionals.

In contrast to the trend examined above, during the pandemic three-year period, the crime of Circumvention of incapacitated persons also decreased by 10% compared to the previous period. In this case, opposite to the trend that characterized the pandemic period regarding crimes of personal injury and family mistreatment, the fact of having gone out of the house “less” somehow prevented situations of financial abuse, which was often, in fact, perpetrated by individuals outside the family unit who defrauded the elderly by exploiting their loneliness or conditions of physical or mental inferiority.

Based on all the judgments examined (in some cases there are multiple charges ascribed to the same defendant), in the period 2020–2023, the conviction of the perpetrator occurred in 59% of cases, while in 41% of cases, the perpetrator was acquitted. The last four-year period was characterized by an increase in convictions (almost 60%) compared to 51% in 2015–2019 and 35% in 2010–2014.

Despite the increase in cases brought to our attention compared to the previous decade seeming to indicate greater awareness of the problem, it is evident that a valid strategy for intervention and management of the phenomenon is increasingly necessary. Also in light of the progressive “aging” of the global population, elder abuse is, in fact, an increasingly widespread social problem, with worrying proportions and characterized by devastating human and economic consequences.

An increase in knowledge related to the characteristics of mistreatment, as well as the mechanisms correlated to it, therefore appears necessary to ensure adequate prevention, early identification, and effective management of the phenomenon under examination.

Physicians and healthcare professionals working in public facilities or assisted living residences therefore occupy a position of fundamental importance for screening and identifying cases of abuse [16, 17].

Mistreated elderly individuals tend to live in often forced isolation; consequently, interactions with healthcare personnel can constitute a unique and essential opportunity for recognizing any signs of abuse in the patient, allowing for the reporting of suspicious cases to the Judicial Authority.

The reporting of suspected mistreatment by a physician represents not only a legal obligation but also a disciplinary duty, dictated by the Code of Medical Ethics itself.

It is, therefore, a competence that should be acquired by all professionals in the healthcare field, and particularly by specialists who more often interact with elderly subjects.

In this regard, the position of the family doctor is of extreme relevance, as they should be able to act as a first “filter,” knowing the patient’s history and their intra-family dynamics in more detail.

The Multidimensional Geriatric Assessment, or Comprehensive Geriatric Assessment (CGA), is certainly the gold standard for an exhaustive evaluation of the elderly person’s health and well-being. Despite this, it is a tool that requires time and specific skills, characteristics that make it unsuitable for use in particularly limited timeframes (visits to the family doctor, emergency departments, or non-geriatric specialists) [9].

Secondly, fundamental help comes from the professional figure of the geriatrician who, knowing in detail the psychophysical changes related to frailty syndrome, is the most competent in identifying any signs of abuse.

Among the figures of considerable importance in this regard are finally emergency room physicians, dermatologists, and orthopaedists, who more often find themselves having to distinguish accidental traumatic physical outcomes from those due to violence and mistreatment.

A series of prevention tools have been proposed, applicable in these contexts, which allow for a quicker, direct or indirect, evaluation of suspected abuse. First, the elderly person should be interviewed separately from family members or the suspected perpetrator of violence, with general questions regarding the safety of the living environment (“do you feel safe where you live?“) and the person responsible for the patient’s care (“Who prepares your food? Who writes the checks?“). Several questionnaires have been proposed for suspected mistreatment: “Has anyone ever hurt you at home? Have you ever been touched without your consent? Has anyone ever made you do things you didn’t want to? Have they ever taken something of yours without asking? Have you ever been reprimanded or threatened? Have you ever signed documents whose meaning you didn’t understand? Are you afraid of anyone at home? Are you often alone? Has it ever happened that someone didn’t help you when you needed it?” [18]. Furthermore, the guidelines drawn up by the ECLM in 2019 for the approach to suspected cases of abuse provide general conduct to be adopted towards the elderly, in order to more easily recognize signs of abuse [19].

If there is a strong suspicion of violence, external testimonies should be sought from sources close to the two subjects (relatives, neighbors, care personnel), in order to obtain further confirmation. The ideal assessment should then include a visit to the elderly person’s home to adequately assess the safety of the living environment and any direct or indirect signs of abuse or neglect [8].

It is fundamental to distinguish normal age-related changes from possible signs of abuse. There are five main health problems that can be misleading, as their symptoms can be confused with those of abuse: skin lesions, unusual bleeding, bone fractures, malnutrition, and anogenital lesions [20, 21].

For better prevention and identification of the problem, it might be useful to:

- Provide a univocal definition of “elder abuse” to establish the true prevalence of the phenomenon and to be able to categorize observed cases within a single definition, thus leaving little room for individual interpretations;
- Establish a standard screening tool, universally applicable across all care settings, regardless of time constraints imposed by the case;
- Educate healthcare personnel to identify, collect, and report any evidence of elder violence to the Authorities;
- Promote the creation of interdisciplinary teams to achieve more rapid and comprehensive management of the problem, integrating the work of various specialists and coordinating it with the intervention of Social Services or Law Enforcement.

However, screening and the prompt identification of abuse situations by healthcare professionals constitute only a small part of the feasible strategies for global prevention of the problem, which can be achieved by addressing the root causes of mistreatment mechanisms, namely its risk factors.

It is therefore clear that all interventions at the social level, designed to provide adequate support to the elderly person themselves as well as to the caregiver responsible for their care, hold even greater significance.

Firstly, it is essential to provide particular attention and support to the elderly, ensuring good social and psychological assistance, so that they can recognize any dangerous situations for themselves, thus being able to know how and to whom to report them.

Finally, psychological and economic support systems should be implemented for families facing the burdensome task of assisting a non-self-sufficient elderly relative. This is, in fact, often a demanding and debilitating task, capable of generating conditions of psychological distress and conflict that inevitably lead to a higher incidence of abuse.

In cases where mistreatment is perpetrated within elderly care homes by healthcare personnel, the underlying reasons for the phenomenon must be sought primarily in the inadequate training of operators and in the work-related stress they often experience.

As preventive strategies in this area, it is highlighted that effective training of facility staff is of fundamental importance, associated with a reduction in workload and an increase in the staff-to-resident ratio.

In conclusion, elder abuse thus constitutes an extremely complex and heterogeneous problem, presenting an infinite number of manifestations and facets, making it difficult to perceive and identify.

Its multifactorial nature also paves the way for various particularly complex management issues: the global prevention of the phenomenon, the early identification of mistreatment cases, the protection of the victim, and the simultaneous recognition of difficulties related to the caregiver are delicate issues that should prompt adequate consideration by public opinion as soon as possible.

Awareness and understanding of the phenomenon therefore represent the most effective tools to recognize and bring to light the submerged part of the “iceberg” and to protect one of the most fragile and valuable resources our society possesses.

## Data Availability

No datasets were generated or analysed during the current study.
